# Strong primary care and patients’ survival

**DOI:** 10.1038/s41598-019-47344-9

**Published:** 2019-07-26

**Authors:** Michel Wensing, Joachim Szecsenyi, Petra Kaufmann-Kolle, Gunter Laux

**Affiliations:** 1 Full professor at Heidelberg University Hospital, Department of General Practice and Health Services Research and Implementation Science, Im Neuenheimer Feld, 130.3, 69129 Heidelberg, Germany; 2 Full professor at Heidelberg University Hospital, Department of General Practice and Health Services Research, Heidelberg, Germany; 3 Pharmacist, senior staff member at AQUA-Institute, Goettingen, Germany; 4 APL Professor at Heidelberg University Hospital, Department of General Practice and Health Services Research, Heidelberg, Germany

**Keywords:** Health services, Health policy

## Abstract

Primary healthcare is the cornerstone of any healthcare system. A major health system reform to strengthen primary care has been implemented in Germany since 2008. Key components include: voluntary participation, intensive management of patients with chronic diseases, coordination of access to medical specialists, continuous quality improvement, and capitation-based reimbursement. The objective of this study was to assess the effect of this reform on survival of enrolled patients. We conducted a comparative cohorts study with 5-year follow-up, starting in the year 2012 in Baden-Wuerttemberg, Germany. Participants were 1,003,336 enrolled patients and 725,310 control patients. A Cox proportional hazards regression model was applied to compare survival of enrolled patients with a composed control cohort of non-enrolled patients, adjusted for a range of patient and physician characteristics. Average age of enrolled patients was 57.3 years and 56.1% were women. Compared to control patients, they had lower mortality (Hazard Ratio: 0.978; 95% CI: 0.968; 0.989). Participation in chronic disease management programs had independent impact on survival rate (Hazard Ratio 0.744, 95% CI: 0.734; 0.753). We concluded that strong primary care is safe and potentially beneficial in terms of patients’ survival.

## Introduction

Strong primary care is characterized by the provision first point of access to healthcare for most people and most health issues, high continuity over time and coordination across healthcare providers, and a strong patient-centered approach^1^. Although insight into the causal mechanisms is limited, epidemiological studies reported on better health outcomes and lower healthcare costs in healthcare systems with strong primary care^2,3^. Despite these benefits, primary care is under pressure across the world, indicated by shortages of primary care capacity and signals of suboptimal performance. Addressing these challenges is therefore high on the political agenda^4,5^.

In Germany, health system reforms to strengthen primary care (‘Hausarztzentrierte Versorgung’) have been implemented in the law (Social Code Book V) in the previous decade. These reforms were designed as voluntary contracts between healthcare insurers and primary care physicians, and voluntary enrollment by patients. These have effectively reformed the healthcare system as they scaled-up intensive management of chronically ill patients, coordination of access to medical specialist care, and participation of primary care physicians in continuous quality improvement activities. Studies reported that these programs increased patients’ use of primary care^6^, reduced number of hospital admissions^7^ and healthcare costs^8^. The aim of this study was to assess the effects of the strong primary care model on patient survival.

## Results

### Description of data

Data on 1,003,336 enrolled patients and 725,310 control patients were available (Table 1). In the population of enrolled patients, the mean age was 57.3 (SD 18.7) years and 56.1% were women. 83.4% had the German nationality, 36.6% were retired, and the mean Charlson-Index for co-morbidity was 1.45. Enrolled patients had been on average nearly 6 years in the program, meaning from one year before start of the observation period of this study. The cohort of non-enrolled patients had slightly different descriptive features, for instance younger average age (54.4 years) and fewer co-morbidities (mean Charlson-Index 1.14).

**Table 1:** Description of patient cohorts.

	Intervention cohort	Control cohort	P-Value
Absolute numbers	1,003,336	725,310	n.a.
Mean age (SD)	57.3 (18.7)	54.4 (19.8)	p < 0.0001
Gender -%women	56.1	56.2	n.s.
Nationality -%German	83.4	81.0	p < 0.0001
Insurance -%Member -%Family -%Retired	52.9 11.6 36.6	54.2 13.7 32.1	p < 0.0001
Comorbidity -Mean Charlson Index (SD)	1.45 (2.10)	1.14 (1.85)	p < 0.0001
Mean number of quarter years in strong primary care program (SD)	23.3 (8.6)	0	n.a.
Mean number of contacts in primary care (SD)	13.65 (11.86)	9.01 (11.08)	p < 0.0001
Mean number of uncoordinated contacts with medical specialists (SD)	2.06 (7.99)	3.37 (9.60)	p < 0.0001
Hospital admissions per 100 patients (SD)	29.0 (79.5)	28.6 (79.1)	p < 0.001
Costs of ambulatory prescribed medication (SD)	1,452.35 (72,751.71)	1,451.63 (64,450.69)	p < 0.001

Legend. All figures on this closed cohort refer to the last year of observation.

### Healthcare utilization

The mean number of yearly contacts in primary care was 13.65, while the number of uncoordinated contacts with medical specialists was 2.06. The average number of hospital admissions per 100 enrolled patients was 29.0 and the average yearly medication costs were 1452 euro. Control patients had partly different healthcare utilization, most particularly fewer yearly contacts in primary care (9.01 on average) and more uncoordinated contacts with medical specialists (3.37).

### Survival

**Figure 1:**
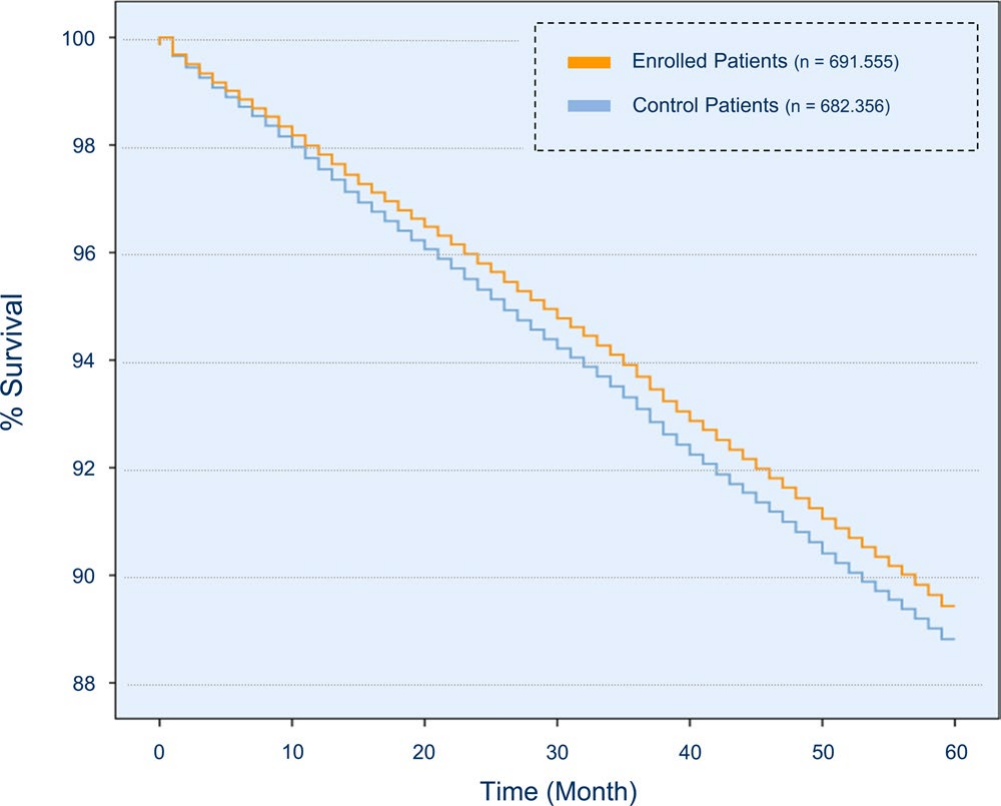
Survival rates, unadjusted.

Figure 1 presents the survival rates of enrolled and non-enrolled patients. Compared to control patients, enrolled patients in the program had lower mortality (Hazard Ratio: 0.978; 95% CI: 0.968; 0.989). Table 2 presents which factors were associated with survival. Participation in disease management programs had independent positive impact on survival (Hazard Ratio 0.744, 95% CI: 0.734; 0.753). An estimated total number of 1,672 lives was saved in the strong primary care arm.

**Table 2:** Predictors of 5-year mortality, adjusted (n = 1, 373, 911).

	Hazard Ratio [95% CI]
Participation in strong primary care delivery model	0.978 [0,968; 0,989]
Sex	1.528 [1.512; 1.544]
Age (in years)	1.076 [1.075; 1.076]
Charlson-Index for co-morbidity	1.213 [1.210; 1.215]
Participation in disease management program	0.744 [0.734; 0.753]
Externally determined need for nursing	1.955 [1.943; 1.968
Living in nursing home	1.109 [1.089; 1.129]
Living in city (as opposed to rural area)	1.023 [1.013; 1.034]

## Discussion

This study in routine healthcare found lower 5-year mortality in patients enrolled in strong primary care as compared to a comparable cohort of non-enrolled patients. Although the small effect should be carefully interpreted, given the non-randomized study design, it was found in a large population in a naturalistic setting and adjusted for many potential confounders. The descriptive data on healthcare utilization suggest that the healthcare delivery model was successfully implemented in enrolled patients. Overall, the study shows that the strong primary care model (including a change to capitation-based reimbursement and structured management patients with chronic diseases) was safe and potentially beneficial in terms of patients’ survival.

Strengths of the study are the inclusion of the total eligible and large study population, the naturalistic setting, and extensive adjustment for potential confounders in the analysis. The influence of patient self-selection was reduced by composing a control cohort on the basis of patients from non-participating physicians; these patients did not have the option to enroll. However, the influence of physician self-selection cannot be ruled out. As the observation period started four years after initiation of the program and high numbers of physicians have joined the healthcare delivery model, it seems plausible that not only early adopters of the innovative program had joined.

Evaluation of the patient-centred medical home, a healthcare delivery model which has many features of strong primary care, found increased quality of chronic disease care and reduced overall healthcare utilization and costs^3,9^. It seems to comprise many components of strong primary care^10^. This study confirms these findings in a healthcare system, which shares several features with the healthcare system in the United States. Our study suggested that participation in disease management – one component of strong primary care - was associated with higher survival. While this subgroup effect should be interpreted carefully, it is supported by a substantial body of research in primary care^11^. The relatively high number of patients’ yearly number of contacts in primary care may facilitate various beneficial activities, such as screening for health problems, monitoring of diagnosed diseases, psychosocial support, and intensive care after hospital discharge. Nevertheless, further research is needed to provide insight into the working mechanisms in strong primary care.

## Methods

### Study design

The study is based on the total population of enrolled patients and a composed comparison cohort in Baden-Wuerttemberg, a state in South-West Germany with about 11 million inhabitants and high enrollment in the strong primary care program. The study has a longitudinal, comparative observational design with five years of follow-up. It was based on routinely recorded data from ambulatory medical practice, derived from administrative databases of the largest regional health insurer and partner of the program (AOK Baden Wuerttemberg). It provides health insurance to about 4.4 million individuals; almost 1.5 million of them and about 4.000 primary care physicians were enrolled in the strong primary care model in 2016. These patients were compared with a composed cohort of non-enrolled patients from the same state. The Heidelberg University Hospital Ethics Committee approved the study (No. S-359/2013) and the study was conducted to relevant regulations and methodological guidance. The presented data and interpretations are a very small part of a comprehensive evaluation report^12^ and a summary version^13^ in the German language. The STROBE reporting guideline for cohort studies was followed in preparing this manuscript.

### Study population

Eligible were individuals aged 18 years or older, living in Baden-Wuerttemberg, at least one visit to the primary care physician in the year of enrollment, health insurance with AOK, no enrollment in other integrated care programs. Patients enter or (after one year) leave the program as they wish. They were included in the intervention arm if they were registered at start of observation (the year 2012) and remained included (without interruptions) until death or end of observation five years later (the year 2016). Patients enrolled in the program provided informed consent before participation. They were compared with a control cohort, comprising of eligible patients in the observed years in the same state and health insurer, who were linked to physicians who did not participate in the program (thus, control patients never had to opportunity to decide on participation). These control patients were linked post-hoc to the primary care physician, whom they had visited in at least 50% of their contacts in primary care. If no such linkage was possible, they were excluded from analysis.

### Primary care model

Enrollment in the healthcare delivery model is a free choice for both patients and physicians. The physician receives additional secured reimbursement for each enrolled patient (capitation instead of fee-for-service). Key component of the program include: improved access to primary care for patients (shorter waiting times, absence of out-of-pocket payment for medication); more comprehensive coverage through extended medical training by physicians; gate-keeping in access to secondary care; improved information transfer between primary and secondary care; structured disease-management for diabetes, asthma/COPD, coronary heart disease run in the primary care practices; continued medical education is coordinated and independent of industry; data-drive quality improvement; and use computerized decision support for drug prescribing. Details of the program have been described elsewhere^6^.

### Measures

The primary outcome in this study was overall survival of patients as documented in the administrative database of the health insurer in the years 2012 to 2016. Patient age, sex, urbanity, recorded morbidities, nursing need, nursing home were documented. The Charlson-Index was calculated to summarize comorbidity^14^. We also documented selected indicators of the fidelity of the healthcare delivery model: numbers of contacts in primary medical care, non-coordinated contacts with medical specialists, hospital admissions, and costs of ambulatory prescribed medication. All measures were based on data in administrative databases and similarly defined as in previous evaluations^6,7^.

### Data-analysis

Differences between the two cohorts (presented in Table 1) were statistically tested. The statistical analysis focused on the comparison of survival rates between the intervention and control cohorts, using Cox proportional hazards regression models. Given the self-selection of physicians and patients into the intervention arm, we first identified predictors of physician participation (considering age, gender, urban/rural, practice size) and predictors of patient participation (considering age, gender, urban/rural, Charlson-Index, and participation in disease management programs) in (separate) logistic regression analyses. Baseline predictors with significant impact (P < 0.01, two-sided test) were included in the primary analysis as co-variables. It may be noted that statistically significant between-group differences at baseline do not necessarily imply confounding in the estimation of intervention effectiveness, because the variables may not be prognostic for outcomes^15^. In order to check the proportional hazards assumption^16^, the ASSESS-statement of SAS procedure PHREG^17^ was used; this showed that it was fulfilled. In the final regression models, effects were expressed with 95% confidence intervals. The Population Attributable Fraction (PAF)^18^ was used to calculate “saved lives” within the observation period, adjusted for confounders. PAF is the estimated fraction of all cases that would have occurred (death) if there had been no enrolment in the strong primary care program, adjusted for measured confounders. The analyses were performed using SAS 9.4 (SAS Institute Inc., Cary, North Carolina, USA).

### Ethics approval

Ethics approval was provided by the Heidelberg University Hospital Ethics Committee (No. S-359/2013).

## Data Availability

AOK Baden-Wuerttemberg can be contacted for secondary analyses of their administrative data.
